# Large-scale tissue clearing (PACT): Technical evaluation and new perspectives in immunofluorescence, histology, and ultrastructure

**DOI:** 10.1038/srep34331

**Published:** 2016-09-29

**Authors:** Peter H. Neckel, Ulrich Mattheus, Bernhard Hirt, Lothar Just, Andreas F. Mack

**Affiliations:** 1 Institute of Clinical Anatomy and Cell Analysis, University of Tübingen, Tübingen, Germany

## Abstract

Novel techniques, like CLARITY and PACT, render large tissue specimens transparent and thereby suitable for microscopic analysis. We used these techniques to evaluate their potential in the intestine as an exemplary organ with a complex tissue composition. Immunohistochemistry, light sheet-, and confocal scanning-microscopy enabled us to follow complex three-dimensional structures, like nerve fibers, vessels, and epithelial barriers throughout the entire organ. Moreover, in a systematic electron microscopic study, we analyzed the morphology and preservation of tissue on ultrastructural level during the clearing process. We also connect tissue clearing with classical histology and demonstrate that cleared tissues can be stained with Hematoxylin-Eosin and Heidenhain’s Azan stain, suggesting potential use in histopathology. These experiments showed that a neutral pH during the clearing process results in much better preservation of tissue ultrastructure and standard stainability. Volume changes of specimens were monitored and quantified during the course of the protocol. Additionally, we employed the technique to visualize the enteric nervous system and the epithelial barrier in post mortem human gut preparations. Our data show the high potential of tissue clearing throughout different tissue types supporting its usefulness in research and diagnosis, and contribute to the technical discussion of ultrastructural tissue-retention.

In order to evaluate the three-dimensional organization of complex organs and tissues, biologists are largely restricted to the use of serial sections and post-hoc reconstruction of the cytoarchitecture. This methodology is highly time consuming, requires a lot of computational effort, and is prone to sectioning artifacts. Recently developed optical sectioning methods like confocal optics, two-photon and light sheet microscopy can overcome some of these obstacles but are limited by the diffraction properties of tissue components. Therefore, clearing and microscopical evaluation of intact tissues is a very compelling and challenging approach. In the late 19th/early 20th century, the German anatomist Werner Spalteholz presented “Aufhellungspräparate”, organs and tissues that were translucent by a combination of H_2_O_2_ dependent bleaching and refractive index matching^1^. However, due to the rather limited microscopical technology at that time, these pioneering steps did not leave a deep impact on the scientific landscape. Fuelled by the development of optical sectioning methods, a number of novel techniques evolved especially in the last five years as recently reviewed by Richardson and Lichtman^2^. These new tissue clearing approaches make use of refractive index matching^3^,^4^, hyperhydratation^5^, lipid solving^6^,^7^,^8^, and hydrogel-embedding^9^,^10^,^11^,^12^, thereby yielding better tissue conservation and allowing for the detection of intrinsic fluorescent protein and/or immunohistochemical staining.

New tissue clearing techniques were largely established and have already been frequently used for the central nervous system [like CLARITY (http://clarityresourcecenter.com/CLARITY_papers.html)]. However, other organs with more complex composition of different tissues (i.e. epithelial, connective, muscle and nerve tissue) such as the gastrointestinal tract with more extensive extracellular matrix have only sparsely been analysed. An exception is the just recently published whole-body clearing preparations with PARS (perfusion-assisted agent release *in situ*) and PACT (passive clarity technique) by Yang *et al*.^11^, ACT-PRESTO by Lee *et al*.^10^, and advanced CUBIC by Susaki *et al*.^13^ as well as two other studies introducing improvements to the original CLARITY protocol^14^,^15^. However, all of these reports performed only general lectin and nuclear staining as proof-of-principle studies in gut preparations.

In CLARITY and PACT protocols proteins are hypothesized to be fixed to an acrylamide scaffold with paraformaldehyde, while lipids are washed out by an amphiphilic substance (e.g. sodium dodecyl sulfate). As membrane lipids are the main cause for light diffraction in tissues, lipid elution renders the specimen translucent. The aim of the present study was not only to test this hypothesis but also to probe the usability of CLARITY/PACT-based tissue clearing methodologies in an organ with a complex composition of different tissue types. The intestine offers a diverse architecture of muscle, connective and nervous tissues, as well as an epithelium with highly fragile histo-architecture like villi or mesenteries. We used specific antibodies commonly used in gastroenterological research to visualize these different tissue components as well as different cellular structures in murine and human specimens. We furthermore used adapted CLARITY/PACT to address the question of changes that occur in tissues during the clearing process. We therefore analyzed specimens using post-clearing paraffin embedding and sectioning with classical histological techniques (Hematoxylin-Eosin, Heidenhain’s Azan stain), thereby linking the novel clearing methods to widely used gold-standards in histopathology and diagnostics. In addition, we show by transmission electron microscopy (TEM) the impact of hydrogel-embedding and tissue clearing on lipid membranes and other structures in TEM ultra-thin sections.

## Results

### Clearing of intestinal tissue

**Figure 1:**
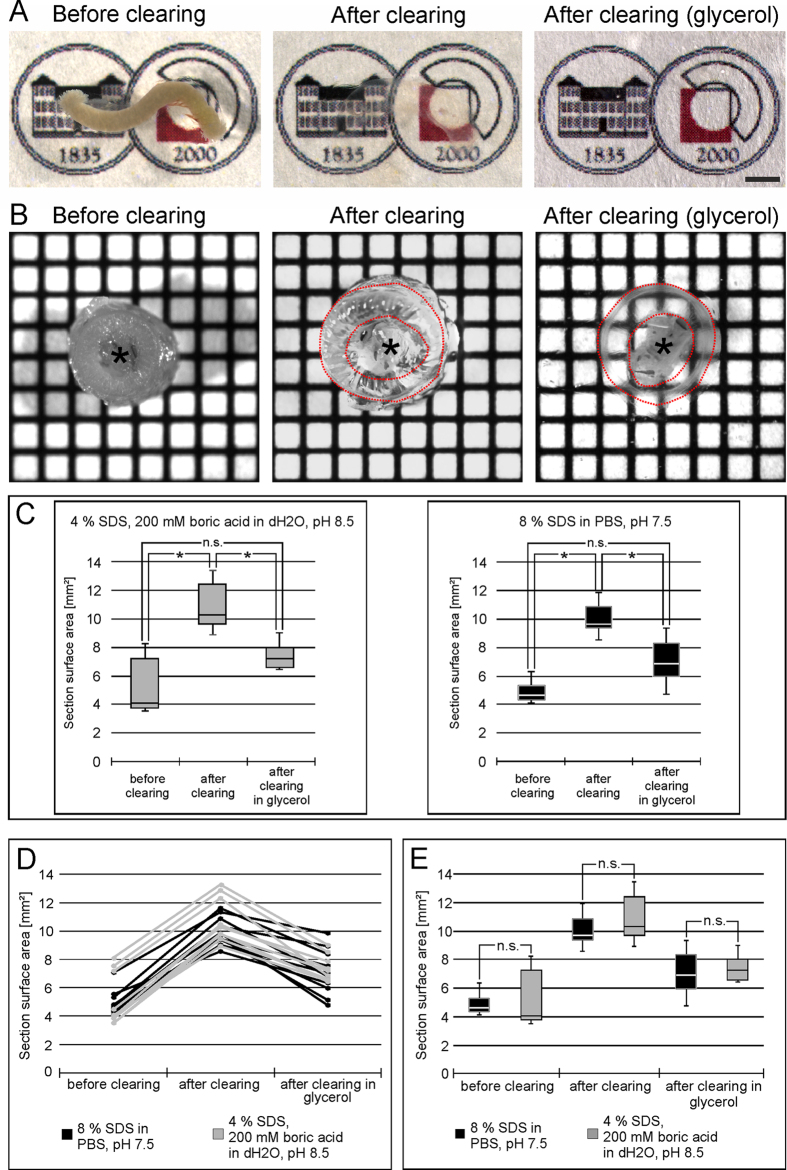
Clearing of gut segments and swelling analysis. (**A**) Shows three images of different steps in the clearing protocol of the same gut segment: After fixation with hydrogel monomer solution submerged in PBS (**left**), after passive clearing submerged in PBS (**middle**), and after clearing submerged in 80% glycerol (**right**). The logo of the authors’ institute underneath the tissue becomes visible during the process illustrating the level of transparency reached. **Scale bar:** 2 mm. In (**B–E**) the swelling of 2 mm thick transversal slices of adult mouse gut is shown. In (**B**) the same slice (**red dotted line**) is depicted before clearing (**left**), after clearing (**middle**), and after clearing submerged in 80% glycerol (**right**). The scale pattern in the background shows 1 × 1 mm squares. (**C–E**) Quantifies the swelling of the specimen during clearing and the successive shrinking after glycerol incubation. (**C**) Illustrates the swelling and shrinking within one group treated with “CLARITY” (**gray**) or “PACT” (**black**) clearing solution. The asterisks indicate statistical significance (Kruskal-Wallis One Way Analysis of Variance on Ranks with post-hoc all pairwise multiple comparison (Dunn’s Method), n = 20, p = <0, 001). In (**D**) the measurement of every single specimen can be followed during the clearing process indicating the uniformity of results from sample to sample. In (**C**) we compared the “CLARITY” (**gray**) or “PACT” (**black**) clearing solutions and found no significant difference between the respective groups concerning the swelling and shrinking effects (Kruskal-Wallis One Way Analysis of Variance on Ranks, n = 20). The asterisk indicated the lumen with residual chyme.

We used the small intestine of young mice to test the applicability and potential of clearing techniques. As shown in Fig. 1A, a typical gut specimen cleared completely within 12–14 days making the logo of the authors’ institute visible through the completely transparent gut. Similar to the clearing process described by Yang *et al*.^11^ we used a passive clearing approach at 55–60 °C. For further analyses we applied two different clearing agents, the original CLARITY clearing solution consisting of 4% (wt/v) sodium dodecyl sulfate and 200 mM boric acid in dH_2_O at pH 8.5 (“CLARITY solution”^9^) and the PACT solution (8% (wt/v) sodium dodecyl sulfate in PBS at pH 7.5 (“PACT solution”^11^). We compared swelling and shrinking of specimens during the clearing process (Fig. 1B–E), clearing velocity, staining behavior, and ultrastructure after clearing (see below).

For swelling and shrinking analyses we used 2 mm thick transversal slices of adult mouse gut. To measure the change in size, we quantified the area of the section surface. As Fig. 1B strikingly shows, there was a distinct increase of tissue size during the incubation in clearing solution, and shrinkage after submerging the specimens in 80% glycerol. These changes were statistically significant, whereas no significant difference was detected between sample sizes before the clearing process compared to samples after the glycerol incubation (Fig. 1C), indicating that tissue volume decreased close to its starting point despite an intermediate swelling during the procedure. This is also supported by the representation of every single sample tested in Fig. 1D. Interestingly, there was no significant difference in the swelling and shrinking behavior of specimens cleared with “CLARITY” or “PACT” solution (Fig. 1E). Neither did we detect any difference in the velocity of the clearing process between specimens incubated in CLARITY solution and PACT solution (data not shown).

### Specific Immunohistochemistry of different tissue types in the cleared intestine

We applied commonly used antibodies to visualize different tissues in the gut especially the enteric nervous system, vascular system, smooth muscle layers, and gut epithelium. Therefore, we present antibody stains against the markers β III tubulin, HuC/D, tyrosin hydroxylase for the enteric nervous system, cytokeratin, ZO-1, serotonin, aquaporin-4, for the epithelial tissue, smooth muscle actin (SMA) for smooth muscle cells and CD31 for blood vessel endothelial cells. All antibody stainings are summarized in Table 1, including those antibodies that did not provide any specific labeling in cleared gut tissue.

Since entire gut pieces were cleared and stained, large image stacks were generated which are difficult to present in a classical printed paper layout using two-dimensional figures. We therefore highly encourage the reader to view the provided images in high resolution and watch the supplementary videos to fully appreciate the three-dimensional tissue composition.

### Visualizing the enteric nervous system completely throughout large scale specimens

**Figure 2:**
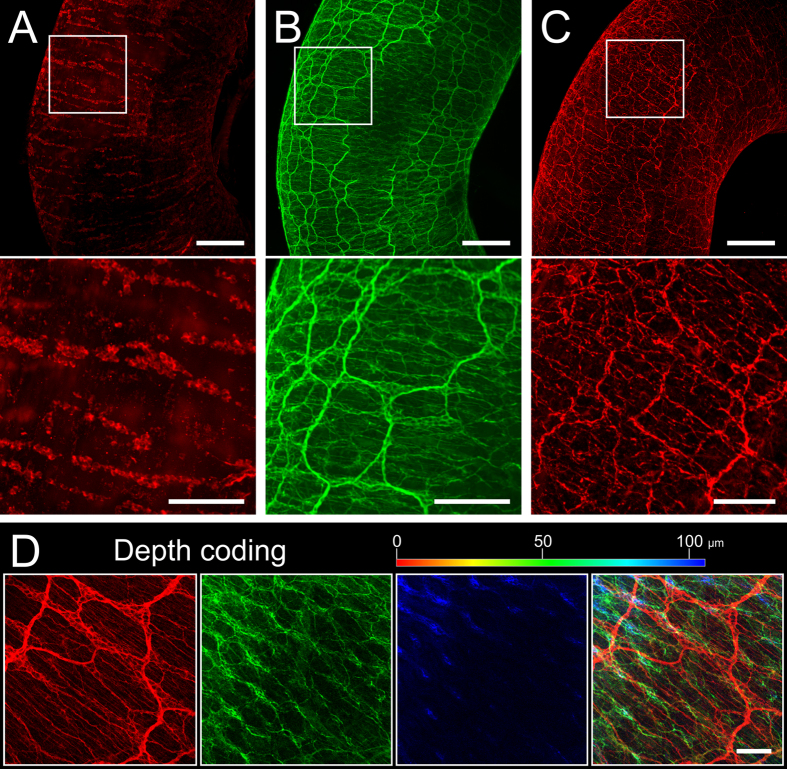
Visualizing the enteric nervous system. In A-C the ENS of different cleared gut specimens are stained for the pan-neuronal markers HuC/D (**A**) and TUJ-1 (**B**), and the catecholaminergic neuron marker TH (**C**). The micrographs below show image details from the upper panels as indicated by the white frames. Images are reconstructions of image stacks acquired on a light sheet microscope with a 5x/0.16 objective in z-step intervals of 8.1 μm over a range of 1863 μm (**A**), 2034 μm (**B**), and 1708 μm (**C**). (**D**) shows a TUJ-1 staining through the gut wall recorded in a confocal image stack (10x/0.3 objective, pinhole = 1 Airy Unit). We used a depth-dependent color-code to visually dissect layers of the ENS plexus from outside (red) to inside (blue) showing the different morphologies of the ENS network in different layers of the gut wall; the right panel shows the overlay. **Scale bars:** (**A–C**) upper panels 500 μm; (**A–C**) lower panels 200 μm; D 100 μm. The specimen depicted in (**B**) is visualized in Supplementary video 1, the z-stack shown in (**D**) can be seen in Supplementary video 2.

We established three major antibody stainings used in ENS research on cleared specimens, namely the pan-neuronal markers β III tubulin (TUJ-1) and the RNA-binding protein HuC/D, as well as tyrosine hydroxylase (TH) specifically found in catecholaminergic neurons. Figure 2 clearly shows the distinct staining of nerve cell somata (HuC/D: Fig. 2A), and nerve fibers (TUJ-1: Fig. 2B,D, Supplementary Video 1, and TH: Fig. 2C). Especially the TUJ-1 staining makes it easy to identify the dominating myenteric and submucous plexus. The staining was equally strong throughout the entire gut wall, which allowed for detailed evaluation of the ganglia and branching pattern of the two major ganglionated plexus as well as fine fibers in the villi. The difference in the two plexus can best be seen in the depth coding approach visually dissecting the morphology of the enteric neural networks in different layers of the gut wall (Fig. 2D, original image stack in Supplementary Video 2). These data open a new way of evaluating the structure of the ENS and possible changes occurring in the course of e.g. aging or disease throughout the gut wall.

### Tissue Clearing reveals the structural complexity of the mesentery-intestinal interface

**Figure 3:**
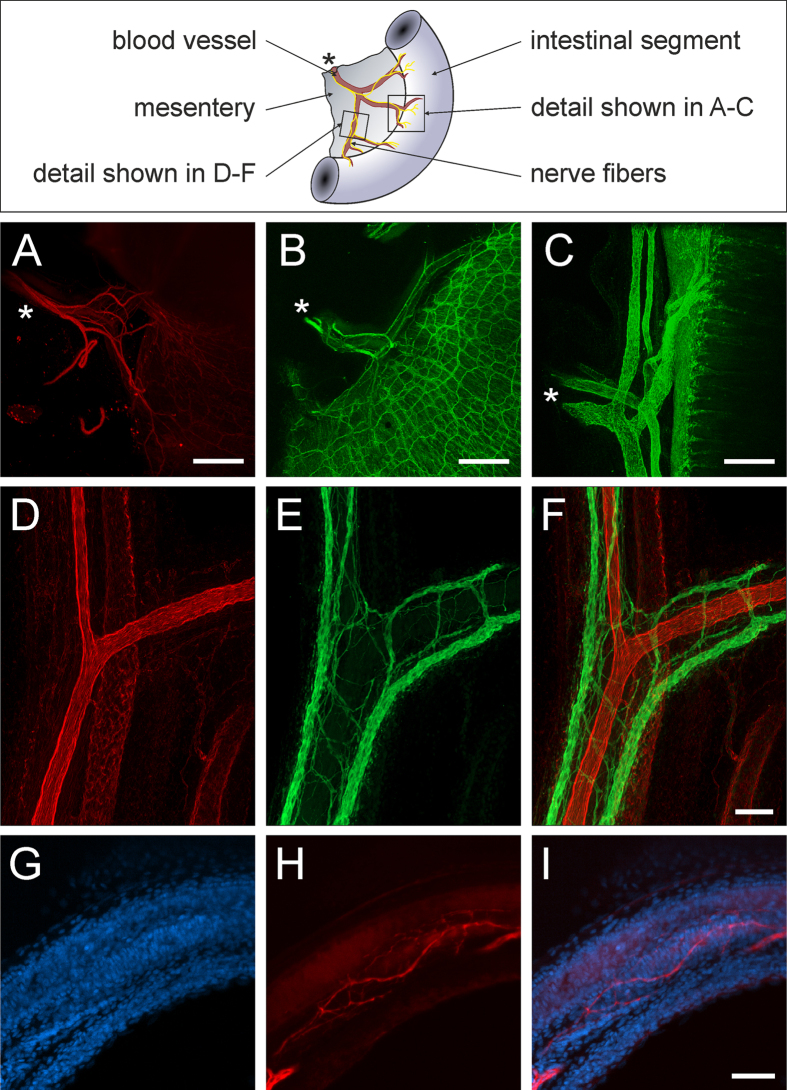
Nerve fibers and vessels in the mesentery. The top panel shows a schematic view of the gut preparation with mesentery attached. (**A**) Shows a reconstruction of a light sheet image stack (5x/01.16 objective, total z-range 2100 μm) of catecholaminergic nerve fibers stained for TH. (**B**) Is reconstructed from a confocal image stack (5x/0.16 objective, pinhole = 1 Airy Unit, z-range 852 μm) nerves stained for the pan-neuronal marker TUJ-1. (**A**,**B**) Illustrate the integration of nerve fibers in the mesentery (asterisk) into the intramural neuronal networks of the gut. (**C**) Depicts blood vessels stained with the endothelial marker CD31 and shows the branching of vessels coming from the mesentery (asterisk) and fine capillaries in the villi (confocal image stack 10x/0.3 objective, z-range 1047 μm). (**D–F**) Show the innervation of blood vessels in the mesentery ((**D**) CD31, (**E**) TUJ-1, (**F**) Merge). (**G–I)** Identifies the very fine sympathetic nerve fibers of arterioles by specific catecholaminergic staining. **Scale bars:** (**A–C**) 500 μm; (**D–F**) 100 μm; (**G–I**) 100 μm. The specimen depicted in (**B**) is visualized in Supplementary video 3, and (**C**) in Supplementary videos 4 and 5. The blood vessels in (**D**–**F**) are shown in Supplementary video 6.

The intestine is connected to the retroperitoneum by connective tissue bridges, so called mesenteries, in which blood vessels and nerve fibers run connecting the intraperitoneal gut segments to the cardiovascular and nervous system. As we were interested in the innervations and blood supply of the gut, we investigated the interface between the mesentery and the intestine and stained for sympathetic (TH: Fig. 3A,E) and pan-neuronal (TUJ-1: Fig. 3B) nerve fibers, and endothelial cells in blood vessels with CD31 (Fig. 3C). In Fig. 3 it becomes clear, how mesentery nerve fibers join the ganglionated plexus of the ENS (Fig. 3A,B, Supplementary Video 3) and how the mesentery blood vessels branch within the gut wall to supply every villus in the tunica mucosa (Fig. 3C; see Supplementary Videos 4 and 5 to follow the blood vessels). Figure 3D–F shows arterioles and venules within the mesentery stained with the endothelial marker CD31. By staining for TUJ-1 we were able to visualize the elaborate innervations of the muscular tunica media of the arterioles. TH and DAPI stainings in Fig. 3G–I distinctly identified these fine innervations as sympathetic fibers of the mesenteric arterioles. Displaying the mesentery and intact gut in one preparation is a major improvement in visualizing the neuronal and vascular interface between the alimentary tract and its extrinsic supply, rendering CLARITY-based immunochemistry a promising tool going beyond previous whole mount preparations.

### 3D villus architecture revealed by specific epithelial staining

**Figure 4:**
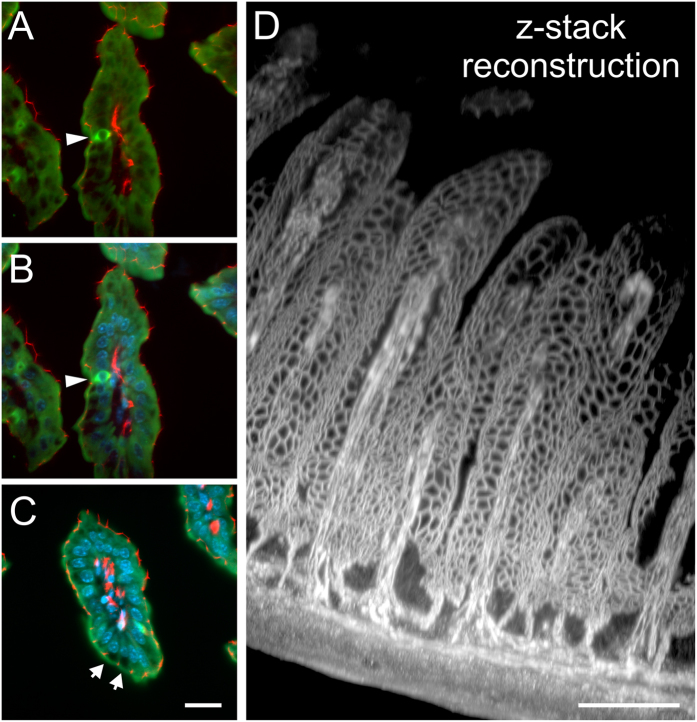
Staining the epithelium. (**A–C**) Show cross sections through gut villi stained with the epithelial marker cytokeratin (green) and tight junction associated protein ZO-1 (red, **A**) in combination with a nuclear DAPI stain (blue; **B,C**). Cytokeratin is expressed throughout the cytoplasm sparing the nuclei. Some cells show intense cytokeratin stain (arrow heads), whereas goblet cells are devoid of it in the apical half of the cell (arrows in **C**). The ZO-1 stain allows the localization of the apical junctional complex and thus reveals the epithelial surface organization. Images and reconstruction from a light sheet image stack recorded with a 20xClr Plan-Neofluar 20x/1.0 Corr nd = 1.45 objective, z-steps 2 μm, total z-range 370 μm. Stack subsets displaying an entire villus are shown in Supplementary video 7, and through a goblet cell in Supplementary video 8. (**D**) Depicts the projection of a partial stack through the entire intestinal wall illustrating the meshwork of the terminal bar complex in the epithelium and capillary endothelium. Due to the high resolution in the z-axis provided by the light sheet microscope the detail of this image can best be appreciated in a fly-around animation shown in Supplementary video 8. **Scale bar:** 100 μm.

We used antibodies against cytokeratin, the tight junction associated protein ZO-1, aquaporin 4 (AQP-4), and serotonin to evaluate the histology of the gut epithelium in cleared preparations. Figure 4 shows villi, stained with ZO-1 and cytokeratin together with DAPI (Fig. 4A–C). Cytokeratin reliably stained the cytoplasm of all epithelial cells, with few strongly positive cells throughout the epithelium and no detectable prevalence in crypt or villus tip (arrow heads in Fig. 4A,B). Goblet cells could also be identified by the cytokeratin stain as there was no fluorescent signal in the apical cytoplasm, where secretory vesicles are kept (Fig. 4C, arrows). ZO-1 staining shows the borders of epithelial cells, more specifically the terminal bar complex over the entire villus and at the endothelial cells of the blood vessels as seen in Fig. 4A–C. This allowed an evaluation of the terminal bar meshwork especially in the 3D reconstruction (Fig. 4D) and animation (Supplementary Videos 7 and 8, and a partial image stack through a goblet cell, video 9). Note also the capillary loops in each villus. Additionally, we stained for serotonin to identify the enteroendocrine cells (Supplementary Video 10). Interestingly, those cells were intensely stained, whereas varicosities of serotonergic neurons are hardly visible. We reasoned, this is due to the big difference in serotonin concentration found in endocrine cells compared to the very small reservoir of serotonin stored in synaptic boutons. We also used an antibody against AQP-4, a water channel known to be found on the basolateral side of epithelial cells^16^ (Supplementary Figure 1, Supplementary video 11). Collectively, our results show that protein epitopes in the epithelium are conserved in the cytoplasm and plasma membrane. Furthermore, delicate structures like the villus tip are preserved throughout the fixation process.

### Staining post mortem human gut specimen

As murine gut samples are considerably smaller than human specimens, we used “PACT solution” to clear full thickness gut wall preparations collected from an 82-year-old donor, 9 h post mortem (Supplementary Figure 2A). The specimens became translucent and could easily be mounted between two cover slips (Supplementary Figure 2B,C), although the thick collagen meshwork especially of the tela submucosa did not clear completely and showed considerable autofluorescence. Nevertheless, antibody stainings against the neuronal marker TUJ-1 impressively revealed the structure of the myenteric plexus, subserosal neurons, and fine nerve fibers running within the muscle layers of the tunica muscularis (Supplementary Figure 2D). Furthermore, using anti-ZO-1 stainings we visualized the terminal bar complex of the epithelium (Supplementary Figure 2E). Together, these data show that despite the drawbacks due to collagen, tissue clearing is applicable to human gut samples and thereby underlines its potential in diagnostic or histopathology.

### Cleared tissue histology in paraffin sections–a link to classical histology

**Figure 5:**
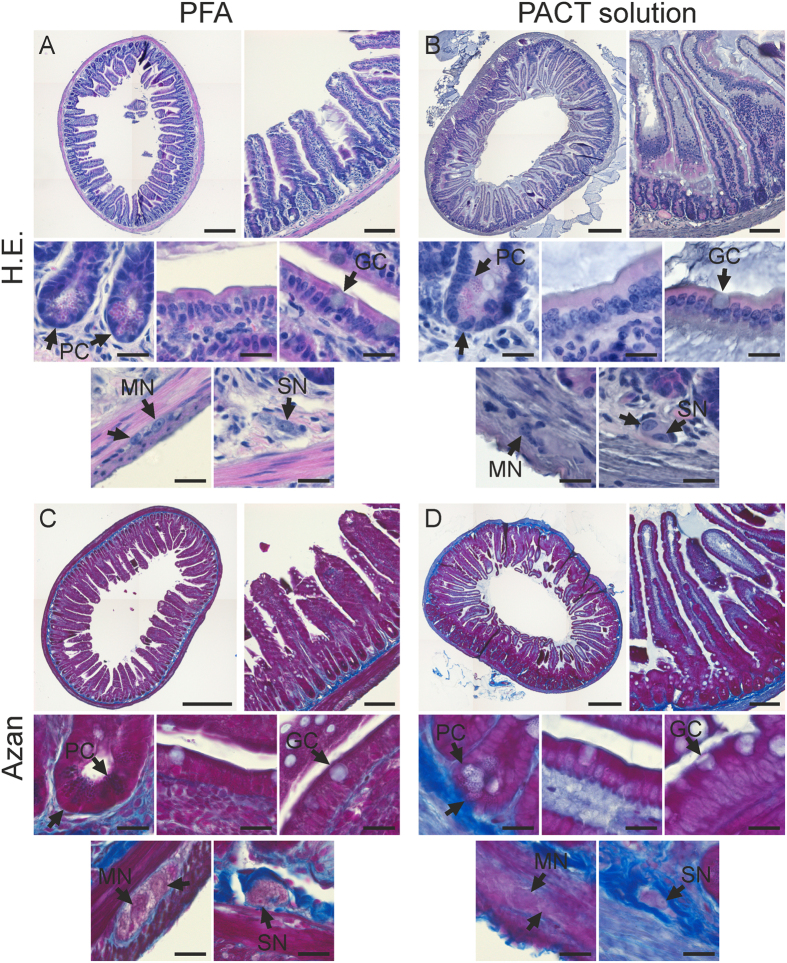
Classical histology of cleared tissue. Hematoxylin-Eosin and Heidenhain’s Azan stain were carried out on transversal paraffin sections of the gut. The specimens were previously either cleared with PACT solution (**B,D**) at neutral pH or fixed with 4% PFA (**A,C**). The uppermost images in each panel show an overview over the entire section (left) and a more detailed image of the entire gut wall (right). High resolution images of paneth cells in the crypts (PC), enterocytes, goblet cells (GC), myenteric (MN) and submucous neurons (SM). The overall tissue retention is equally good in both preparations. Moreover, the acrylamide embedding obviously offers protection to the tips of the villi. **Scale bars:** entire gut cross sections 500 μm; gut wall overviews 200 μm; high magnification images 20 μm.

Having shown that a variety of antibody cell labelling is well compatible with the passive clearing method we wanted to test the general tissue quality after clearing for two reasons: We aimed to test first for the preservation of tissue and cell structure, and second for the compatibility with medical histopathology, since pathologists generally have tremendous knowledge and experience in evaluating sections prepared with classical routine stains. We therefore embedded previously cleared intestinal specimens in paraffin and subsequently performed H.E. and Heidenhain’s Azan staining. As shown in Fig. 5, both staining methods worked for PACT solution cleared tissues nearly as well as for standard 4% PFA fixed tissues used as controls. However, in gut pieces cleared with the original CLARITY solution the staining did not work reliably or was unspecific (Supplementary Figure 3). It is conceivable that the high pH destroys or masks basophilic and acidophilic structures that classical stainings rely on.

Nevertheless, when using the PACT solution, stainings were specific, and different tissue- and cell types could be identified (Fig. 5). Although the red stain of eosin was less intense in previously cleared tissues, smooth muscle cells, ENS neurons, goblet cells, paneth cells, and enterocytes with microvilli were clearly distinguishable and their structure was well preserved. Additionally, we found that villi tips and their epithelium were better retained in the cleared tissue than in the 4% PFA fixed tissue (Fig. 5B,D). We hypothesize that the acrylamide hydrogel in the lumen embedded and protected the filigree villi from erosion. In fact, this hydrogel coat can still be seen in between the villi in Fig. 5B,D. Furthermore, we did not detect any cracks or shrinking artifacts on the borders of different tissues.

In conclusion, clearing with the 8%-SDS solution at pH 7.5 keeps the tissue compatible for post-hoc classical pathological diagnosis.

### Preservation of ultrastructures in cleared gut tissue depends on clearing solution

**Figure 6:**
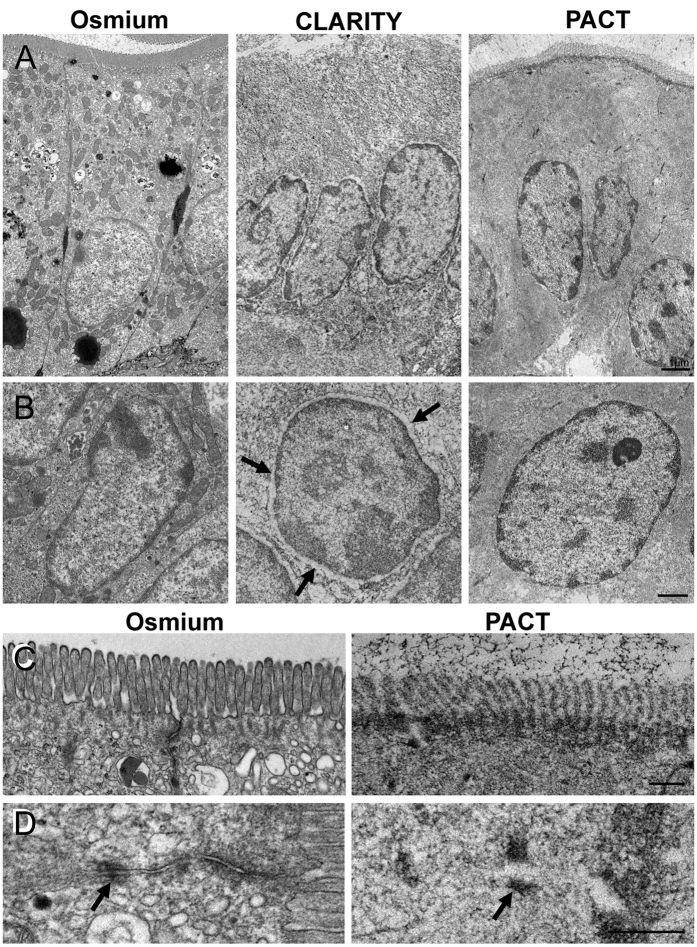
Transmission electron microscopy of cleared tissue. Shown are ultrathin sections of specimens fixed and contrasted with osmium only (**A–D left**), and of tissues cleared previously with CLARITY solution (**A,B middle**) and PACT solution (**A–D right**). In **A**, overviews of enterocytes are depicted, below are high resolution images of enterocyte nuclei (**B**), apical microvilli (**C**) and subapical junctional complex (**D**). Lipids were washed out of all cleared specimen indicated by the lack of stained membranes. Tissues cleared in CLARITY solution exhibited shrinking artefacts and an enlarged perinuclear cleft (**B, arrows**) as well as a loss of fine structures especially at the apical part of the enterocytes. Tissues treated with PACT solution retained fine structural features like actin filaments in microvilli (**C left**) desmosomes (**D, arrows**) although the membranes are lost. **Scale bars:** A and B 1 μm, C and D 500 nm, for all images in the same row, respectively.

To extend the evaluation of tissue quality we investigated previously cleared samples on the electron microscopical level and compared them to standard treatment with respect to the gut-specific ultrastructural features. We especially focused our analysis on the epithelium as its structure is well known and is moreover highly sensible to specimen treatment. In Fig. 6A enterocytes are depicted which were treated with standard TEM methods as control (Fig. 6A left), after passive clearing with CLARITY solution (Fig. 6A middle), and after passive clearing with PACT solution (Fig. 6A right). In the panels below, nuclei of enterocytes with the respective treatments are shown (Fig. 6B). From these data it becomes obvious that membranes are entirely washed out by the clearing process. This effect makes it especially difficult to contrast the tissue, as membranes normally are heavily stained by osmium and serve as the main cell structure for orientation in electron microscopy. We further found that samples cleared with the CLARITY solution exhibited more tissue damage, especially at the apical part of the enterocytes (Fig. 6A middle), as well as shrinking artifacts at the nucleus, as the large perinuclear cleft indicates (Fig. 6B middle). We did not observe these effects in specimens cleared with the PACT solution, further supporting that the better tissue preservation is due to the neutral pH of the clearing solution.

We additionally looked at fine structural features in more detail, namely microvilli and cell-cell contacts, and compared PACT solution cleared tissue with control specimens (Fig. 6C,D). From Fig. 6D it is obvious that proteins within the desmosomal complex remain fixed in the tissue and preserve ultrastructural location. Furthermore, actin filaments located in the microvilli can be recognized as well as the terminal cytoskeleton net below the apical membrane (Fig. 6C). Together our data show that ultrastructural cytoarchitecture remains preserved throughout the clearing process with the CLARITY solution. For the first time we provide a direct comparison between the quality of specimens previously cleared and specimens processed by standard treatment for electron microscopy.

## Discussion

In this report we provide evidence that a variety of cellular epitopes including cytoskeletal, cytoplasmic, or membranous proteins can be immunocytochemically labelled in cleared whole-gut pieces and imaged throughout these specimens. Modern clearing methods allow for deep penetration of staining compounds and light into the specimens, while being at the same time gentle to the tissue^2^,^3^,^4^,^5^,^6^,^9^,^11^. In CLARITY and PACT, the high degree of permeability is hypothesized to be due to the wash-out of membrane lipids which are the major components of diffusion barriers and light refraction in tissues, whereas the rest of the biomolecules are stabilized and fixed to an acrylamide scaffold^9^,^11^. In the present study, systematic ultrastructural analyses of cleared specimens of the gut as well as different histological methods strongly support this theory on different levels of histo-architectural organization.

Our findings demonstrate on the light- and electron microscopical level that cellular and subcellular structures are retained during the process of tissue clearing. The previously reported hypothesis that membrane lipids are washed out by the incubation in SDS-solutions by ultrastructural analyses is strikingly demonstrated in Fig. 6C where the membrane coat of microvilli is missing in PACT cleared tissue on the ultrastructural level. In fact, this effect makes it quite difficult to image these specimens in electron microscopy, as osmium contrast largely relies on interactions with membranes. However, we were able to show a tissue architecture preserved well enough to identify the fine structure of actin filaments in the microvilli or sub- and transmembrane proteins in cell-cell junctional complexes like desmosomes. The original article on CLARITY in 2013^9^ reports that electron microscopic evaluation of previously cleared tissue is possible. However, as this work was proof of principle, a systematic comparison of cleared and control tissue was not provided. The protein wash-out assays carried out by Yang and co-workers^11^ are consistent with our data strongly indicating that tissue clearing at a neutral pH does not change the concentration or the positioning of proteins in the sample.

This is also in concert with our finding that other tissue components remain well preserved at a neutral pH, as H.E. and Heidenhain’s Azan staining revealed. Futhermore, antibody-stains for intracellular epitopes, like TH, HuC/D, or serotonin, as well as cytoskeletal structures (e.g. TUJ-1, cytokeratin) confirm these results. The most astonishing finding is the preservation of membrane-associated epitopes like ZO-1, AQP4, and CD31, since these proteins were still detected at the correct location despite the lipid wash-out.

On the tissue level our results further support a good retention of the specimens so that different cell-types remain distinguishable in classical histology and immunohistochemistry due to specific protein expression and preserved morphology. We also could visualize complex three-dimensional cytoarchitectures, like capillary loops in single villi, or the complicated way mesentery nerves and vessels integrate into the intramural networks. Furthermore, tissue clearing also allowed us to assess fine structures within the gut wall, like the filigree patterning of the ganglionated plexus of the ENS.

To optimize staining conditions, we compared the two most frequently reported clearing solutions, namely 4% (wt/v) sodium dodecyl sulfate and 200 mM boric acid in dH_2_O at pH 8.5 (CLARITY solution^9^, (Chung *et al*. 2013)) and 8% (wt/v) sodium dodecyl sulfate in PBS at pH 7.5 (PACT solution^11^). While we could not detect any difference between samples cleared with the respective solutions in terms of clearing-time, clearing-efficiency, tissue expansion, and immunohistological staining, there were obvious discrepancies in classical histology and ultrastructure. In fact, we were not able to reliably stain sections previously cleared with the CLARITY solution with H.E. or Heidenhain’s Azan stain. Likely, the high pH of the CLARITY solution masked or destroyed acidophilic or basophilic structures that these procedures rely on. Moreover, TEM experiments revealed shrinking artifacts of nuclei and structural aberrations especially at the filigree apical side of the gut epithelium. Again, it is conceivable that the high pH leads to this kind of artifacts by proteolysis and nucleic acid hydrolysis. We want to point out that this effect may only occur in combination of high pH with the high temperature and long incubation times needed for passive clearing. Nevertheless, we show that post-hoc ultrastructural analyses can be carried out in specimens cleared at a neutral pH.

It is noteworthy that gross volume changes during the clearing process are not different for the two clearing solutions used. Therefore, we assume that intracellular loss of structures is caused by a different mechanism than specimen volume changes. Despite the swelling and shrinking of tissue volume, we did not detect any cracks between the different tissue layers of the gut in classical histology, indicating that size changes are a predictable and even process.

A recent study performed tissue clearing without embedding into acrylamide gel^17^. Arguing that the suggested cross-linking by acrylamide plays only a minor role in tissue preservation the authors show that sufficient clearing is achieved by SDS alone for brain tissue. The authors show H.E. staining and scanning electron microscopic images of the cleared specimens, but it remains to be shown whether cellular and tissue integrity is maintained during this procedure in other organs as well.

Although we successfully tested several different antibodies specific to neuronal subtypes, endothelial cells, epithelial cellular and subcellular components, a few antibodies against commonly used epitopes, e.g. NOS1, cKIT, and ChAT, did not show a specific staining. We want to emphasize that antibodies which did not work for our staining protocol might not be completely inapplicable to CLARITY-based immunohistochemistry, but could be used with adaptations in incubation temperature or duration, fixative or acrylamide concentration. Another improvement of immunolabeling could be achieved using novel methods like described by Li and colleagues^18^ or even alternative fixation protocols like SWITCH^19^.

Moreover, we were not able to see any difference in the quality of immunostainings in tissues cleared with CLARITY or PACT solution on cellular level (data not shown).

It was one aim of this study to assess the potential of modern tissue clearing for research on the alimentary tract as an example for an organ with several tissue interfaces. We used the murine small intestine as a model to test different well known and established antibody stainings to assess the different tissues of the gut, like the enteric nervous system, blood vessels, or the epithelium, including subcellular structures using modern light sheet imaging technology. We visualized different neuronal subtypes, the ganglionated plexus of the ENS, and fine neuronal protrusions in villi and around blood vessels. This offers tremendous advantages for structural analyses of the ENS (e.g. during aging or to characterize phases of disease) without stretching and sectioning artifacts as well as the assessment of the fine innervation of effector tissues (e.g. muscles, glands, or immune cells). In contrast, the current standard in whole mount preparations of the gut can introduce slicing or preparation artifacts and suffers from a lack of standardization^20^,^21^. In fact, two pioneering studies using post-staining refractive index matching already foreshadow how tissue clearing will improve our understanding of the complex histo-architecture of the intestine^22^,^23^.

Furthermore, we successfully evaluated the neuronal and blood vessel histology in the mesentery. Thus, we can directly follow blood vessels and nerve fibers from the mesentery to the nerve plexus and gut villi, respectively. We want to stress the fact that the interface between the mesentery and the gut is only sparsely explored, as this filigree structure is often destroyed during preparation and difficult to image. We therefore see a high potential of acrylamide embedded clearing methods for this field of research. Especially the evaluation of the vascular structure of the gut parenchyma in cancerous or inflamed (chronic or acute) states offers a high potential for future studies.

Tissue clearing allowed for detailed evaluation of the epithelium and the terminal bar complex on a large area without cutting or sectioning the tissue. This makes it possible to investigate the changes of the epithelial barrier and will be helpful in future studies on microbiome-host interactions or microbial entry routes of infections.

Modern immunohistochemical methods in combination with tissue clearing methods has put forward the idea of personalizing histopathological diagnostics using specific antibodies^24^. This could help to phenotype tumors by immunostaining against e.g. surface epitopes specific to cancer cells. Clearly, the time-consuming procedure of PACT does not suggest this method for a daily routine, yet we envision that improvements in the methodology might render it more useful in diagnostics, for example identifying blood vessel structures in tumors. Our results close the gap between the novel phenotyping technologies of tissue clearing and the well-established classical histopathology and diagnostic experience. These results therefore enable pathologists to use novel tissue clearing methods for personalized phenotyping and still make use of their experience with classical sectioning methods to identify and classify pathologies.

In this context we were interested if acrylamide-based tissue clearing could be scaled up to larger human gut specimens, as it was shown for human brain tissues before^9^. Indeed, we here show immunostainings of neuronal networks in the tunica muscularis as well as epithelial structures in human full thickness gut wall samples. It is noteworthy however that the elaborate connective tissue (especially collagen within the tela submucosa) does not clear and therefore hampers microscopic analysis (Supplementary Figure 2): This felt-like connective tissue makes it impossible to scan though the entire gut wall, at least with conventional confocal imaging. In addition, especially collagen exhibits a marked autofluorescence. These obstacles can already partly be overcome by two-photon microscopy and second harmonic imaging^25^. The latter one even uses the special optical behavior of collagen to unravel changes in connective tissue architecture in chronically inflamed biopsies^26^.

Collectively, our data clearly show the high potential modern tissue clearing offers to the field of gastroenterology and enteric neuroscience. It furthermore contributes to the methodological development of these new techniques and substantiates the reliability of SDS-based lipid washout with systematic ultrastructural data. Moreover, we present the possibility to use previously cleared tissue for classical histological staining procedures, thereby bridging the gap of novel tissue clearing approaches to pathohistological diagnostical standards.

## Material and Methods

### Animals

C57BL/6N mice (Charles River Laboratories Germany, Sulzfeld, Germany) were used for breeding and experiments. Animals were handled and all methods were carried out in accordance to the institutional guidelines of the University of Tübingen, which conform to international guidelines. All experimental protocols were approved by the regional committee (Regierungspräsidium Tübingen).

Early postnatal mice (P0-P5) were decapitated; adult mice (8 weeks old) were anesthetized with CO_2_ followed by cervical dislocation and the whole gut was removed. The mesenteria were gently severed, so that the intestinal convolute could be unfolded leaving the distal mesentery ends attached. 1–2 cm long gut segments were then cut and the lumen was perfused with hydrogel monomer solution (see below) using a 0.30 diameter insulin syringe (BD Biosciences, Heidelberg, Germany). Thereby, chyme and mucus (which do not clear), and bile salts (which are still yellow after the clearing process) can be washed out. For best results, perfusion had to be done gently to avoid harming the epithelium by sheer forces or rupturing the gut wall by applying too much pressure.

### Human tissue samples

Our study includes samples collected from one cadaver donated to the Institute of Clinical Anatomy and Cell Analysis in Tübingen by a male volunteer aged 82. The body donor gave his informed consent in concert with the declaration of Helsinki to use his cadaver for research purposes. The procedure was approved by the ethics commission at the Medical Department of the University of Tübingen (Project Nr. 237/2007 BO1). Samples were taken 9 hours *post mortem* (Supplementary Figure 2).

### Fixation and hydrogel-embedding

Murine tissues were fixed in hydrogel monomer solution (HMS) composed of 4% (wt/v) acrylamide, 4% (wt/v) paraformaldehyde, and 0.25% (wt/v) VA-044 Initiator (WAKO, cat. No 017-19362) in PBS. The lumen of entire gut segments was perfused with HMS before the specimen was submerged in HMS for 2 days at 4 °C.

After fixation, individual specimens were put into 50 ml tubes with sufficient HMS to cover them entirely. The tubes were placed in a vacuum oven (Thermo Scientific Heraeus, Hanau, Germany) and degassed at 13.3 kPa for 15 min. Afterwards, the oven was heated to 37 °C for 3 h at 13.3 kPa to start polymerization. After the gel solidified, we used paper tissues to gently remove gel rests from the tissue.

### Tissue clearing

We used passive clearing for lipid wash-out, as clearing the entire intestinal specimens can be achieved in less than two weeks. Therefore, we used either a clearing solution consisting of 4% (wt/v) sodium dodecyl sulfate and 200 mM boric acid in dH_2_O at pH 8.5 (in the following called ”CLARITY solution”^9^), or 8% (wt/v) sodium dodecyl sulfate in PBS at pH 7.5 (in the following named “PACT solution”^11^). Specimens were incubated in 40 ml of either clearing solution at 60 °C on a rotator. Clearing solution was exchanged every 3–5 days. Clearing process was completed depending on the size of the tissue after 12–14 days. After clearing, the samples were washed twice in PBS with 0.1% (v/v) Triton X-100 for one day.

### Staining and refractive index matching

Cleared samples were blocked with PBS supplemented with 1% (wt/v) bovine serum albumin, 0.25% (v/v) Triton X-100, 0.1% (wt/v) sodium azide, 4% (v/v) donkey serum for 2 days at 37 °C. Afterwards, primary antibodies were diluted in 1% (wt/v) bovine serum albumin, 0.25% (v/v) Triton X-100, 0.1% (wt/v) sodium azide, 4% (v/v) donkey serum in PBS as described in Table 1. Sufficient antibodies were applied so the specimens were well covered by the solution, usually 1–3 ml. Tissues were incubated in primary antibody solution at 37 °C for up to one week, with fresh antibody being added to the solution after 4 days. Next, specimens were washed with 0.1% (v/v) Triton X-100 in PBS for 2 days each at 37 °C. Secondary antibodies were diluted in PBS (Table 1) and incubated just like the primary antibody solution at 37 °C for up to one week. Again fresh antibody was added after 4 days of incubation.

Afterwards, samples were stained with DAPI (1.25 μg/ml) for 12 hours and subsequently washed in PBS 3 times for 5 hours. Then the cleared tissues were incubated in 80% (v/v) glycerol in dH_2_O until the samples were absolutely translucent.

### Paraffin sections and classical histology

To evaluate the tissue maintenance after the clearing procedure and the possibility to use classical histological methods in previously cleared tissue, we processed specimens for paraffin histology and subsequent Hematoxylin-Eosin and Heidenhain’s Azan staining on cleared and standard 4% (wt/v) PFA fixed specimens: After clearing, the tissue was embedded in paraffin using a standard tissue processor (Citadel tissue processor; Shandon-Thermo Scientific, Waltham, MA, USA) for dehydration and transfer into paraffin. Paraffin blocks were sectioned at 3 μm using a microtome (HM355 SS; Microm International, Walldorf, Germany). Sections were placed on Super Frost Plus slides (Microm International, Walldorf, Germany), dewaxed (Xylol for 3 × 5 min) and rehydrated by descending alcohol concentrations (100%, 96%, and 70% for 5 min each) to distilled water. For tissue evaluation, Hematoxylin-Eosin and Heidenhain’s Azan staining were used.

### Microscopy and data acquisition

Specimens were visualized either by conventional confocal or light sheet microscopy. For standard confocal imaging, samples were positioned in a glass bottom petri dish (Fluoro Dish, World Precision Instruments) submerged in a drop of 80% (v/v) glycerol in H_2_O to minimize shifting and moving of the specimen during image acquisition. Human gut samples were mounted as described in Supplementary Figure 2. Image stacks were recorded on a LSM 5 Exciter attached to an AxioImager (Carl Zeiss, Microscopy GmbH, Jena, Germany) with a Plan-Neofluar 10x/0.3 objective or Acroplan 20x/0.4 Corr. For imaging on a light sheet microscope (Lightsheet Z.1, Carl Zeiss, Microscopy GmbH, Jena, Germany), samples were glued to a hook-shaped holder, submerged in 80% (v/v) glycerol in H_2_O, and evaluated with an EC Plan-Neofluar, 5x/0.16 objective, and a Clr Plan-Neofluar 20x/1.0 Corr nd = 1.45 objective.

Images were processed and 3D-reconstructions were rendered using ZEN software (Carl Zeiss).

### Electron microscopy

Preparation of ultrathin sections was performed following standard procedure previously published^27^. In brief: Cleared specimens and unfixed control tissue were postfixed in 1% (wt/v) OsO4 in PBS, and then dehydrated in an ethanol series (50%, 70%, 96%, 100%). The 70% alcohol was saturated with uranyl acetate for contrast enhancement. Dehydration was completed by acetone, followed by propylene oxide. Specimens were infiltrated with rising concentrations of Epoxy embedding medium (Sigma Aldrich, Darmstadt, Germany). Ultrathin sections (60 nm) were cut on a Leica Ultramicrotome (Leica, Bensheim, Germany) and mounted on pioloform-coated copper grids. Ultrathin sections were examined and documented using a LEO 912AB transmission electron microscope (Carl Zeiss, Oberkochen, Germany).

### Statistical analysis

Statistical analysis was carried out using Sigmastat 3.5 software (Systat Software, Erkrath, Germany). Differences between groups were assessed using Kruskal-Wallis One Way Analysis of Variance on Ranks with post-hoc all pairwise multiple comparison (Dunn’s Method). A significant difference was assumed with p-values ≤ 0.05.

## Additional Information

**How to cite this article**: Neckel, P. H. *et al*. Large-scale tissue clearing (PACT): Technical evaluation and new perspectives in immunofluorescence, histology, and ultrastructure. *Sci. Rep.* **6**, 34331; doi: 10.1038/srep34331 (2016).

## Supplementary Material

Supplementary Information

Supplementary Video 1

Supplementary Video 2

Supplementary Video 3

Supplementary Video 4

Supplementary Video 5

Supplementary Video 6

Supplementary Video 7

Supplementary Video 8

Supplementary Video 9

Supplementary Video 10

Supplementary Video 11

Supplementary Video 12

## Figures and Tables

**Table 1:** Antibodies used for immunostaining.

Epitope	Host	Dilution	Supplier
NOS1	Rabbit	1:400	Research & Diagnostic Antibodies No. AS-1657; Lot # 9266
HuC/D	Mouse	1:50	Molecular Probes No. A-21271
Chat	Goat	1:100	Chemicon International No. AB144P
TUJ	Rabbit	1:400	Covance Cat. No. PRB-435P
TH	Mouse	1:400	Immunostar Cat. No. 22941; Lot # 1240001
c-KIT	Rabbit	1:200	DAKO No. A4502
Cytokeratin	Rabbit	1:400	DAKO No. Z0622
SMA	Mouse	1:100	DAKO No. M0851
AQP4	Rabbit	1:100	Santa Cruz Biotechnology Inc. No. sc-20812
ZO-1	Rabbit	1:100	Thermo Scientific Cat. 33-9100; Lot # QC215031
CD 31	Rabbit	1:50	Abcam ab28364
Serotonin	Rabbit	1:400	Immunostar No. 20080
Anti-mouse Alexa 546	Donkey	1:400	Thermo Fisher Scientific No. A-21422
Anti-rabbit Alexa 488	Donkey	1:400	Thermo Fisher Scientific No. A-21206
Anti-goat Alexa 488	Donkey	1:400	Thermo Fisher Scientific No. A-11055
Anti-rabbit Alexa 488	Goat	1:400	Thermo Fisher Scientific No. A-11008
Anti-mouse Alexa 546	Goat	1:400	Thermo Fisher Scientific No. A-11030
