# Supplementary material for: Large-scale tissue clearing (PACT): Technical evaluation and new perspectives in immunofluorescence, histology, and ultrastructure
Source: Sci Rep. 2016 Sep 29;6:34331. doi: 10.1038/srep34331 (PMC5041186; doi:10.1038/srep34331)
Supplement: Supplementary Information [file srep34331-s1.pdf]

Supplementary information

to

Large-scale tissue clearing (PACT): Technical evaluation and new perspectives in immunofluorescence, histology, and ultrastructure

Peter H. Neckel[1]; Ulrich Mattheus[1]; Bernhard Hirt[1]; Lothar Just[1]; Andreas F. Mack[1,*]

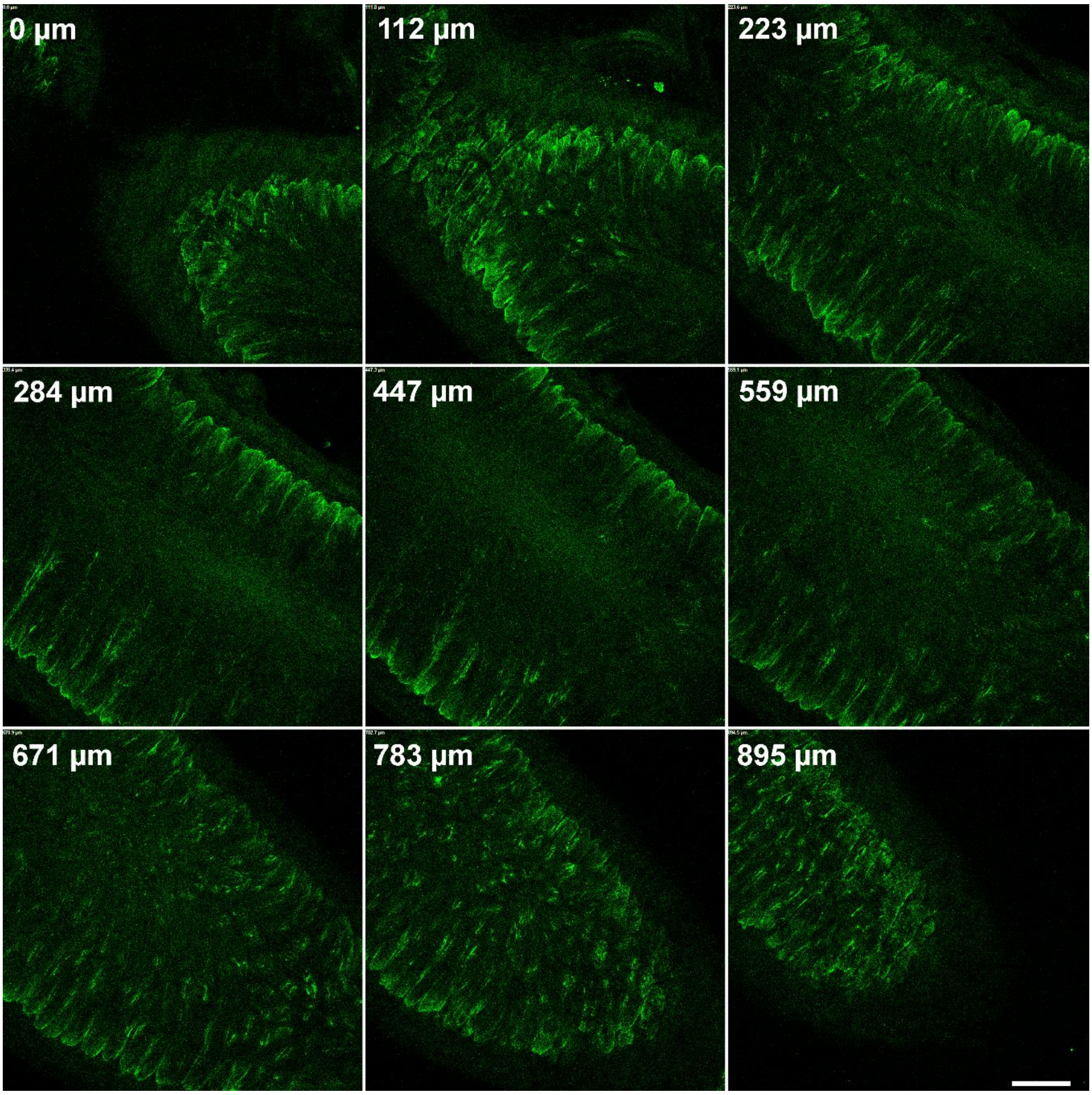

Supplementary figure 1: Aquaporin-4 expression. Shown are selected slices from a confocal z-stack (range as indicated) though a gut sample stained for aquaporin-4 (AQP4). There is a strong immunoreactivity on the basolateral side of enterocytes, especially in the crypt. **Scale bar: 200** µm. The z-stack was recorded with 10x/0.3 objective with 8.6 µm z-step intervals, and is visualized in **supplementary video 11**.

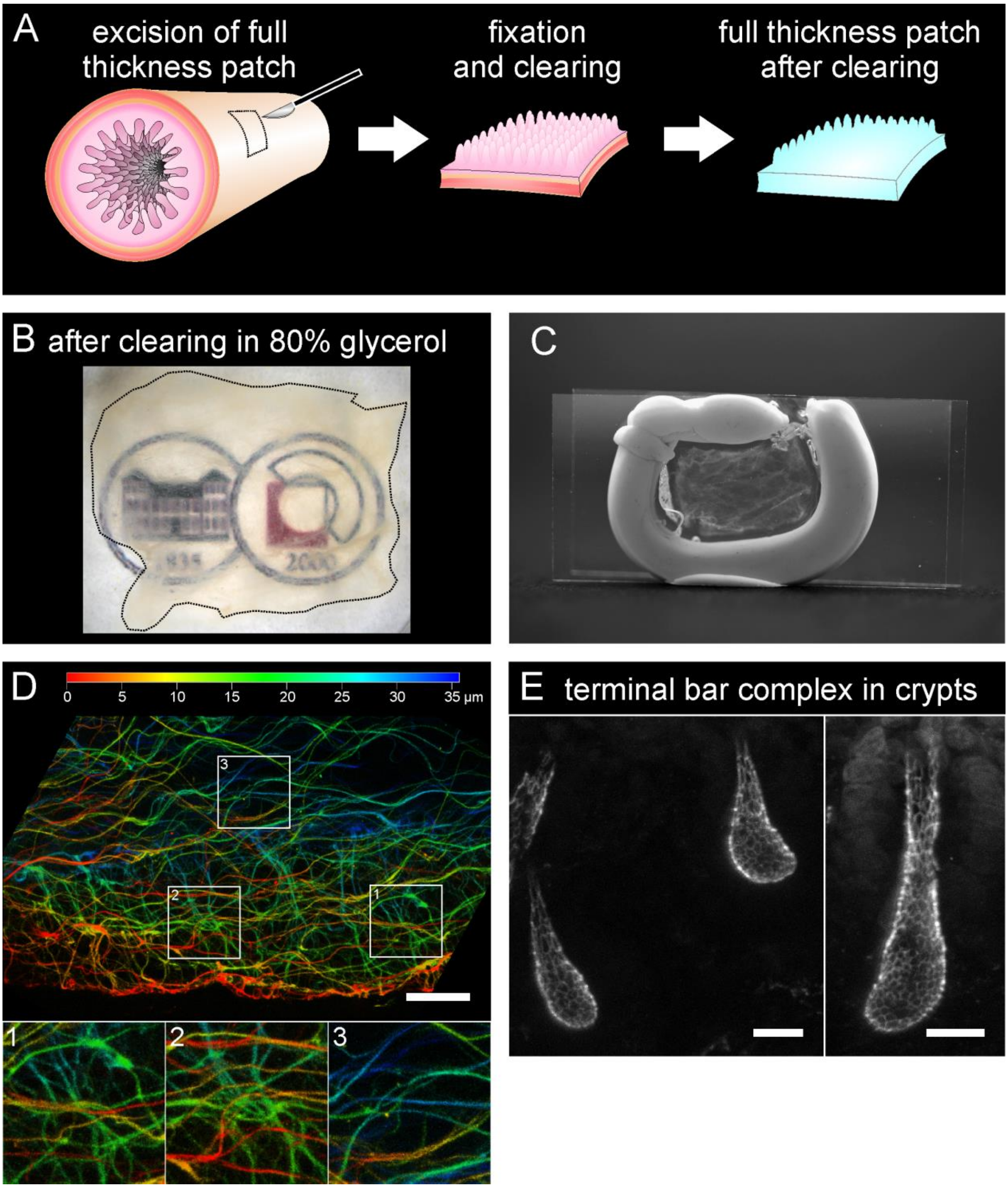

Supplementary figure 2: Clearing full thickness human gut wall. Human samples were collected from a male,82-year-old body-donor, 9 hours post mortem. Full thickness patches of the intestinal wall were excised and cleared as indicated in the scheme in **A**. In **B** one of the samples is shown after the clearing process and submerged in 80 % glycerol in dH_2O. The vast majority of the tissue became transparent, allowing the authors' institutional logo to shine through the specimen. However, the thick collagen meshwork especially in the tela submucosa remains visible. **C** shows an image of how we mounted the sample between two coverslips as it is described by Tomer et al. (2014). We used modeling clay to form a chamber between the cover slips, that was then filled with 80 % glycerol in dH_2O and sealed with ACRIFIX 1-component polymerization adhesive (EVONIK Industires AG, Essen, Germany). **D** shows a depth-coded 3D reconstruction of an image stack of the neuron marker TUJ-1 acquired with

the LSM-5 exciter (Zeiss) using a 10x objective (NA 1.3). The structure of the subserosal neurons (subset 1), the myenteric plexus (subset 2), and fibers in the muscle layers (subset 3) are distinguishable. The z-stack is visualized in **supplementary video 12.** In E the terminal bar complex of crypts is visualized by immunohistochemistry for ZO-1 acquired with the LSM-5 exciter (Zeiss) using a 10x objective (NA 1.3). **Scale bar: D: 40 µm; E: 20 µm**.

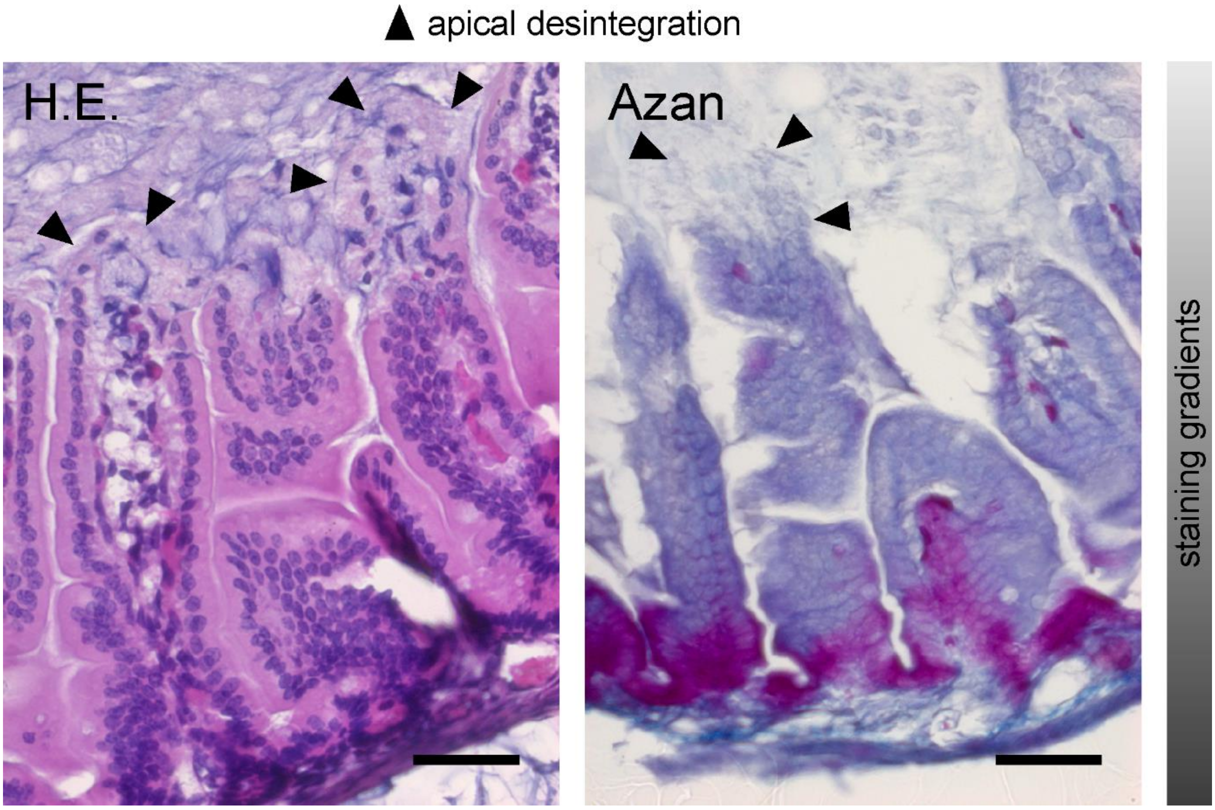

Supplementary figure 3: Classical stainings reveal drawbacks of clearing at a high pH. The micrographs shown H.E. and Heidenhain's azan stainings of transversal sections through the gut wall that were previously incubated in "CLARITY solution" with a pH of 8.5. Unlike the tissues cleared at pH 7.5 (see Fig. 5), the stainings reveal a strong disintegration of the fragile apical villus tips (arrowheads). Moreover, the staining efficiency itself was hampered: We often found dye gradients and unspecific stainings. **Scale bar:** 50 µm.

Supplementary video 1: Enteric nervous system of the entire specimen. The video shows a 3D-reconstruction of the enteric nervous system revealed by staining against tubulin beta III (TUJ-1). The image stack for the reconstruction was acquired on a light sheet microscope with a 5x/0.16 objective in z-step intervals of 8.1 µm over a range of 2034 µm

Supplementary video 2: High resolution neural plexus in the gut wall. The video is a z-stack of TUJ-1-positive cells and fibers of the enteric nervous system. The two major plexus as well as fine fibers around the crypts and in the villi can be distinguished. Confocal stack acquired with 10x/0.3 objective, z-step7.03 µm, z-range 210 µm.

Supplementary video 3: Neural connections in the mesentery. 3D-reconstruction of the interface of mesentery and the gut wall stained for the neural marker TUJ-1. The complex innervation pattern of extrinsic nerves into the enteric nervous system is indicated in the animation generated from a confocal image stack (5x/0.15 objective, z-steps 17 µm, z-range 852 µm).

Supplementary video 4: Blood vessels in the mesentery. Z-stack of endothelial cells (CD31) that line the blood vessels in the mesentery and the gut wall. It is possible to follow the branching pattern of the vessel and fine capillaries in the villi (confocal image stack 10x/0.3 objective,,z-step 8.2 µm, z-range 1047 µm).

Supplementary video 5: Blood vessels in the gut wall. Confocal z-stack of endothelial cells (CD31) that line blood vessels in the gut wall. Note the pattern of the capillary network in the intestinal wall (10x/0.3 objective, z-range 1047 µm).

Supplementary video 6: Blood vessels in the mesentery. 3D-reconstruction of an arteriole within the mesentery. The endothelium is marked with CD31 (red) and the accompanying nerves are stained with TUJ-1 (green). The fine innervation of the tunica media is distinctly visible.

Supplementary video 7: Stainings of the epithelium. Light sheet image-stack of a group of villi stained for the epithelial marker cytokeratin (green), the tight junction-associated protein ZO-1 (red), and DAPI (blue). Besides the junctional complex of enterocytes, endothelial cells are positive for ZO-1. Acquired with a 20xClr Plan-Neofluar 20x/1.0 Corr nd=1.45 objective, z-steps 2µm, z-range as indicated.

Supplementary video 8: The terminal bar complex. 3D-reconstruction of a ZO-1 stain showing the complexity of the terminal bar complex in a group of villi. Reconstructed from a light sheet image stack recorded with a 20xClr Plan-Neofluar 20x/1.0 Corr nd=1.45 objective, z-steps 2µm, total z-range 370 µm.

Supplementary video 9: Goblet Cells. Partial image stack of a villus stained for ZO-1 (red), cytokeratin /green) and DAPI (blue) passing through a goblet cell (arrow), from the same data set as shown in video 7.

Supplementary video 10: Enteroendocrine cells in the epithelium. Light sheet image-stack of an immunostaining for serotonin (green). There is the strong reactivity for serotonin in the

enteroendocrine cells of the epithelium. DAPI is blue, recorded with a 5x/0.16 objective in z-step intervals of 8.2 µm, original stack had a range of 2876 µm), shown is a subset with as range as indicated.

Supplementary video 11: Aquaporin-4 and smooth muscle actin in the gut. Confocal z-stack of an aquaporin-4 (AQP4) and smooth muscle actin (SMA) staining in the gut. AQP4 (green) is restricted to the basolateral side of enterocytes, especially in the crypt. SMA (red) stains the two muscle layers of the tunica muscularis, smooth muscle cells within the villi and the media of arterioles coming from the mesentery (10x/0.3 objective with 8.6 µm z-step intervals, z-range as indicated).

Supplementary video 12: TUJ-1 positive neuronal network in the human small intestine. Depth-coded Z-stack reconstruction of the TUJ-1 positive neuronal fibers and plexus in the human gut wall. Especially the myenteric ganglia and the parallel fibers running along the muscle layers can be appreciated (10x/0.3 objective with 8.6 µm z-step intervals, z-range as indicated).
